# Heat stroke: knowledge and practices of medical professionals in pediatric emergency medicine departments – a survey study

**DOI:** 10.1186/s13584-021-00469-7

**Published:** 2021-06-02

**Authors:** Noy Cohen-Ronen, Ayelet Rimon, Neta Cohen, Tali Capua

**Affiliations:** 1 Pediatric Emergency Medicine, Assuta Ashdod University Hospital, Ashdod, Israel; 2 Faculty of Health Sciences, Ben-Gurion University of the Negev, Beer-Sheva, Israel; 3 Pediatric Emergency Medicine, Dana-Dwek Children’s Hospital, Tel Aviv Sourasky Medical Center Sackler Faculty of Medicine, Tel Aviv University, Weizman Street, 64239 Tel Aviv, Israel; 4 Pediatric Emergency Medicine, The Hospital for Sick Children, Toronto, Canada; 5 Medical Technology and Simulation Center, Tel Aviv Sourasky Medical Center, Toronto, Canada

**Keywords:** Pediatric emergency medicine, Heat stroke, Emergencies, Surveys and questionnaires, Equipment design, Simulation

## Abstract

**Background and importance:**

Heat stroke is a life-threatening condition affecting children worldwide. Rapid cooling remains the most important feature of emergency management. The accepted preferred method of evaporative cooling in the ED as listed by the reference text book endorsed by the Israeli Society of Pediatric Emergency Medicine (PEMI), is actively cooling the patient by spraying him with water and positioning fans to blow air across the body.

**Objective:**

This study aimed to assess Israeli health care workers (HCWs) medical professionals’ knowledge and preparedness of treating heat stroke and recommend policy changes to ensure better treatment based on survey results.

**Design, settings and participants:**

A cross-sectional survey of all HCWs working in an ED that accepts children was conducted. An online questionnaire was utilized to assess heat stroke management practices and available resources in all Israeli pediatric emergency departments (PEDs).

**Main results:**

Data from 208 health care workers was analyzed. Only 30% of the participants reported ever treating a patient with exertional heat stroke. Two scenarios were presented to the participants: motor vehicle-related child hyperthermia (MVRCH) in an infant and an adolescent with exertional heat stroke. One hundred twenty-five (60%) and 83 (40%) participants, respectively, listed cool water with a fan as the primary mode of cooling, which is considered the appropriate preferred method of evaporative cooling in the PED. Certificated pediatric emergency medicine (PEM) physicians answered significantly more correctly regarding both scenarios’ management (*P* < 0.001). Participants who were trained via simulation in the past, answered significantly more correctly regarding the exertional heat stroke scenario (*P* < 0.01), however no difference was found regarding the MVRCH case.

Conclusions: The present study exposes weaknesses in HCW knowledge, PED resources, and published PED policies for appropriate management of children following heat stroke. Our finding emphasizes the importance of both certificated PEM physicians attendance and simulation performance for implementing proper management of patients with heat stroke. A policy change should be performed among the Israeli PED community, with respect to establishing and implementing guidelines for treatment of exertional heat stroke. A future study, following an interventional simulation program is planned to be performed.

## What is known?

- Heat stroke, a life-threatening condition may occur in children and requires rapid cooling to ensure best chance of survival.

## What is New?

- Health care workers knowledge on cooling treatment and practices were found to be varied.
- Certified PEM physicians and simulation may aid in implementing proper management of patients with heat stroke.

## Introduction

Heat stroke is a life-threatening condition clinically diagnosed as a severe elevation in body temperature with central nervous system dysfunction that often includes combativeness, delirium, seizures, and coma [1]. Heat stroke occurs because of high external temperatures or physical exertion, leading to motor vehicle related hyperthermia in children (MVRHC) and exertional heat stroke in athletes in hot environments respectively.

Children left in cars for even short time periods risk death by hyperthermia [2]. In the United States alone, 231 MVRHC fatalities were reported between 1999 to 2007. In 80% of cases, children were left unattended [3]. Similarly in Israel, between 2008 and 2019, over 800 cases of MVRHC were reported, 35 of which ended in a fatality [4]. Israel’s average temperature in the summer months typically range between 35 and 40 degrees Celsius and the temperature in a closed car in the middle of the day can reach 70 degrees Celsius [3]. As a result, the pediatric emergency department (PED) should be prepared for treatment of heat stroke in those conditions.

The prognosis of heat stroke in patients is directly related to the degree of hyperthermia and its duration. Therefore, besides prevention, the most important feature in the treatment of heat stroke is rapid cooling [5]. The method of rapid cooling has long been debated in medical practice. Data mainly from research of exercise-induced hyperthermia suggests that optimal cooling includes submersion in ice or tepid water (1–16 °C), if readily available in the emergency department (ED) [5, 6]. Although several sports organizations suggest using ice-water or cold water immersion for the treatment of patients with heat stroke, basic research studies have shown that evaporative cooling are equally effective [5]. The accepted preferred method of evaporative cooling in the ED as listed by the reference text book endorsed by the Israeli Society of Pediatric Emergency Medicine (PEMI), is actively cooling the patient by spraying him with water and positioning fans to blow air across the body [7]. The historical differences in clinical approach and in the evidence based data, the difficulty to perform prospective studies on children and taking into account that MVRHC is a rare condition in ED, make it a particularly relevant topic to practice using medical simulation. Being a true medical emergency, the approach to treatment of heat stroke must be fast and effective. This study aimed to assess medical professionals’ knowledge of treatment of heat stroke, and ED preparedness to do so, expressed by appropriate supplies and equipment. This study is the first attempt to test the preparedness of PEDs for treating heat stroke in Israel.

## Methods

We conducted a cross-sectional survey to assess hyperthermia management practices and available resources in all Israeli EDs that accept children. An online questionnaire (Hebrew version: https://forms.gle/xyjfehHx1iEu5eJw7, English version: https://forms.gle/Pzdem8oMjuKJJNYz6) was developed.

The questionnaire was designed by the authors after compiling a list of all types of management strategies for heat stroke, by consulting with research literature and with experts of pediatric trauma. The survey questions covered specific procedures and protocols for heat stroke management, based on two clinical scenarios (MVRHC and exertional heat stroke).

Once the online version of the survey was developed in Google forms, it was piloted by two pediatric emergency medicine (PEM) physicians and two head ED nurses, to assess its relevance, its usability, and the total time for completion. The feedback from these participants was incorporated into subsequent modifications.

We contacted the department head and head nurse of each ED or PED in all public hospitals across the country on May 172,020, and asked them to distribute the survey to their staff members. A reminder was sent at least two more times within the study period via WhatsApp and email. Participants were told that the purpose of the survey was to gain a better understanding of treatment of hyperthermia in EDs that accept children. Answers were collected between June 17th and August 17th, 2020. We included any HCWs that were employed in an ED that accepts children. We excluded any uncompleted form.

This study received an ethical waiver from the institutional review board, as it did not use patients or patient data.

## Results

In total 210 questionnaire responses were received. 2 were excluded as they were incomplete. Data from 208 medical professionals (physicians and nurses) aged 25 to 58 were analyzed. Of a total of 22 EDs who care for children in Israel, 20 EDs were represented within the responder group, covering all geographic areas of the country. At the time of the survey there were approximately 40 PEM physicians actively working in PEDS, 20 pediatricians, 400 pediatric residents and 270 nurses. Response rates varied between groups, the highest response rate was the PEM physician group 31/40 (78%), the lowest among nurses 62/270 (23%). The majority of the responders were physicians, largely composed of pediatric residents (40%), and including 15% PEM specialists. The main characteristics of the responders are presented in Table 1 including their experience with real life cases and simulation. Of the 30% who have ever treated a patient with exertional heat stroke, 73% felt the treatment was adequate. Twenty one percent of the responders treated MVRHC in the past, of them 84% reported they felt the treatment was adequate for MVRHC.

**Table 1:** Demographic characteristics and professional details of the responders

Category	Responders (***n*** = 208)
Female gender n, (%)	144 (69)
Age, years mean ± SD	37.5 ± 9
Nurses n, (%)	62 (30)
Physicians n, (%)	146 (70)
PEM specialists	31 (15)
Other physicians	115 (55)
- pediatric residents	83 (40)
- pediatricians working in ED	13 (6)
- PEM fellows	19 (9)
Past experience n, (%)	
Treatment of exertional heat stroke	63 (30)
Treatment of MVRHC	44 (21)
Participation in heat stroke simulation	58 (28)

*SD* standard deviation, *PEM* pediatric emergency medicine, *MVRHC* motor vehicle related hyperthermia in children

When presented with a scenario of an infant with MVRHC, 125 (60%) listed cool water of any temperature with a fan as the primary mode of cooling, which is considered the preferred method of evaporative cooling in the PED. Thirty nine (19%) responded cooling with ice packs, 27 (13%) responded with ice bath, 13 (6%) cold intravenous fluids and one requested peritoneal lavage with cold fluids.

When given a scenario on exertional heat stroke, 83 (40%) listed cool water of any temperature with a fan as the primary mode of cooling which is considered the preferred mode of cooling. Sixty eight (33%) responded cooling with cold intravenous fluids, 30 (14%) responded with ice packs, 10 (5%) responded with ice bath, and 14 (7%) requested peritoneal lavage with cold fluids.

Knowledge of management was assessed for differences between the responders’ specialty and for influence of participation in simulations (Table 2). PEM physicians were more likely to answer correctly when asked what the treatment would be for a MVRHC (26/31 PEM physicians answered correctly vs. 58/115 non-PEM physicians, *p* = 0.00001). Similarly, PEM physicians were more likely to answer correctly when asked about the treatment for exertional heat stroke (20/31 PEM physicians answered correctly vs. 27/115 non-PEM physicians, *p* = 0.0001).

**Table 2:** Knowledge of management according to the physician’s specialty

Category	PEM physicians (***n*** = 31)	Other physicians (***n*** = 115)	***P*** value
Evaporative cooling for MVRHC n, (%)	26 (83.9)	58 (50.4)	0.00001
Evaporative cooling for exertional heat stroke n, (%)	20 (64.5)	27 (23.5)	0.0001

*PEM* pediatric emergency medicine, *MVRHC* motor vehicle related hyperthermia in children

The responders who participated in a hyperthermia related simulation had a higher percentage of correct answers when asked about the exertional heat stroke scenario (34/58 vs. 49/150 *P* = 0.0009). When considering treatment logistics in heat strokes scenarios, of the 125 responders who wished to use a fan, 21 stated they didn’t have a fan, 13 didn’t know where the fan was stored and 41 weren’t sure if they had a fan. Three respondents answered they would use ice in the resuscitation of one or both scenarios but did not know where the ice was located.

## Discussion

This study describes the variability in both theoretical and practical knowledge in treating heat stroke in Israeli EDs. It also identifies the differences between PEM physicians and non-PEM physicians and highlights opportunities for improvements including changes in policy. While good basic knowledge and strategies are reported available in most EDs, there are some important deficits that can be easily implemented to ensure more effective treatment of heat stroke.

A relatively low number of participants in our survey reported ever treating hyperthermia. The medical teams’ limited exposure to heat stroke cases, require educational programs such as medical simulation to be developed. The goal should be to improve both theoretical and practical knowledge and to increase level of comfortability among medical teams, with heat stroke management algorithm and cooling techniques.

Simulation-based training has been shown to be an effective tool that provides a controlled learning environment in which to practice a wide range of clinical scenarios [8–10]. Simulation programs have been shown to make a significant improvement in specific scenarios, especially for scenarios that are rare and critical, such as pediatric cardiac arrest patients [11]. In our survey, a low number of physicians and nurses reported having ever participated in any simulation of hyperthermia (28%). This group was found to have significantly higher rates of acceptable management of the teenage exertional heat stroke scenario, but not in the infant MRVHC scenario. This may be the result of the low rate of simulation participation, resulting in lack of true impact of simulations in our population.

Evaporative cooling was the most frequently selected primary mode of cooling in both scenarios, however it wasn’t the management preferred in the majority of responses in the exertional heat stroke scenario. Despite traditional teaching that intravenous fluids cooled may precipitate arrhythmias, 33% of responders choose this answer. As expected, certified PEM physicians had significantly higher rates of correct management in both scenarios, compared to non-PEM physicians. The importance of having a PEM physician on site is well documented [12].

We recognized not only the knowledge gap in terms of choosing the right cooling method, but also practical barriers in treatment logistics. Personnel were not sure if they have fans in the ED or where they are located. It is extremely important for the medical personnel to be familiar with the equipment in the ED and its operation. All of these goals can be achieved by solidifying a country wide protocol and participating in medical simulations, which provide both theoretical knowledge and practical tools.

The present study had several limitations. A sampling bias may have occurred with regards to the proportion of responders. PED teams are very heterogeneous and vary in number from center to center. Despite surveying all Israeli EDs which accept children, the compared groups were small and response rate varied. As a result, the low statistical power has a reduced chance of detecting a true difference in practice. Lastly, we had no details about the timing, the type and quality of the simulations that responders participated in, causing the influence of past simulation experience to be unequal. Further research should be performed with a pre and post simulation assessment of a uniform heat stroke scenario.

## Conclusions

The present study highlights shortcomings in the understanding and care of children with heat stroke. Policy change should be urgently made by the Israeli PEM community, via establishment and implantation of appropriate guidelines for treatment of exertional heat stroke. A national protocol written in collaboration with the Israeli Ministry of Health, Israeli Association of Emergency Medicine and other stakeholders should be urgently written. Moreover, an interventional educational program composed of simulations should be established. Based on our experience, HCWs should be mandated to attend simulations of such cases at least once every 2 years. Finally, resources should be allocated to PEDs to have both the equipment needed and the personnel with an emphasis on the presence and guidance of certified PEM physicians. Personnel should have regularly guided sessions as to where they may find resources needed to treat children suffering from hyperthermia.

## Data Availability

All data and material may be made available upon request.

## Abbreviations

ED: Emergency department
MVRCH: Motor vehicle-related child hyperthermia
PEDs: Pediatric emergency departments
PEM: Pediatric emergency medicine
